# The effects of resistance training to near failure on strength, hypertrophy, and motor unit adaptations in previously trained adults

**DOI:** 10.14814/phy2.15679

**Published:** 2023-05-05

**Authors:** Bradley A. Ruple, Daniel L. Plotkin, Morgan A. Smith, Joshua S. Godwin, Casey L. Sexton, Mason C. McIntosh, Nicholas J. Kontos, Jonathan P. Beausejour, Jason I. Pagan, Juan P. Rodriguez, Daniel Sheldon, Kevan S. Knowles, Cleiton A. Libardi, Kaelin C. Young, Matt S. Stock, Michael D. Roberts

**Affiliations:** ^1^ School of Kinesiology Auburn University Auburn Alabama USA; ^2^ School of Kinesiology and Rehabilitation Sciences University of Central Florida Orlando Florida USA; ^3^ Department of Physical Education Federal University of São Carlos São Carlos Brazil; ^4^ Biomedical Sciences Pacific Northwest University of Health Sciences Yakima Washington USA; ^5^ Edward Via College of Osteopathic Medicine Auburn Alabama USA

**Keywords:** failure, motor unit, resistance training, strength

## Abstract

Limited research exists examining how resistance training to failure affects applied outcomes and single motor unit characteristics in previously trained individuals. Herein, resistance‐trained adults (24 ± 3 years old, self‐reported resistance training experience was 6 ± 4 years, 11 men and 8 women) were randomly assigned to either a low‐repetitions‐in‐reserve (RIR; i.e., training near failure, *n* = 10) or high‐RIR (i.e., not training near failure, *n* = 9) group. All participants implemented progressive overload during 5 weeks where low‐RIR performed squat, bench press, and deadlift twice weekly and were instructed to end each training set with 0–1 RIR. high‐RIR performed identical training except for being instructed to maintain 4–6 RIR after each set. During week 6, participants performed a reduced volume‐load. The following were assessed prior to and following the intervention: (i) vastus lateralis (VL) muscle cross‐sectional area (mCSA) at multiple sites; (ii) squat, bench press, and deadlift one‐repetition maximums (1RMs); and (iii) maximal isometric knee extensor torque and VL motor unit firing rates during an 80% maximal voluntary contraction. Although RIR was lower in the low‐ versus high‐RIR group during the intervention (*p* < 0.001), total training volume did not significantly differ between groups (*p* = 0.222). There were main effects of time for squat, bench press, and deadlift 1RMs (all *p*‐values < 0.05), but no significant condition × time interactions existed for these or proximal/middle/distal VL mCSA data. There were significant interactions for the slope and *y*‐intercept of the motor unit mean firing rate versus recruitment threshold relationship. Post hoc analyses indicated low‐RIR group slope values decreased and *y*‐intercept values increased after training suggesting low‐RIR training increased lower‐threshold motor unit firing rates. This study provides insight into how resistance training in proximity to failure affects strength, hypertrophy, and single motor unit characteristics, and may inform those who aim to program for resistance‐trained individuals.

## Graphical abstract

Limited data exist examining how resistance training to failure affects applied outcomes and single motor unit characteristics in previously trained individuals. Here, we show that training near failure versus not training near failure for 5 weeks similarly increases strength and does not affect muscle hypertrophy. However, training near failure increases lower‐threshold motor unit firing rates, and further research is needed to determine the significance of this finding.

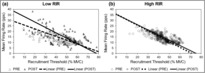

## 1 INTRODUCTION

It is well known that resistance training promotes increases muscle strength and mass and that these outcomes are conferred through neural and musculoskeletal adaptations (Sartori et al., 2021; Skarabot et al., 2021). For decades, it has been suggested that resistance training performed to failure can maximize strength gains and muscle hypertrophy (Drinkwater et al., 2005; Nobrega & Libardi, 2016). It is commonly thought that trained individuals, particularly bodybuilders and strength‐trained athletes, benefit most from resistance training to muscle failure. Trained individuals can tolerate high training stresses, and it has been suggested that resistance training to muscle failure might provide an extra stimulus to increase muscle mass and strength (Zatsiorsky & Kraemer, 2006). Although a recent meta‐analysis suggests that resistance training performed to momentary muscular failure is not superior to non‐failure resistance training for strength gains and muscle hypertrophy (Grgic et al., 2022), these meta‐data contained trained, untrained, young, and elderly subjects. Notably, the sub‐analysis consisted of only two studies in trained participants, which speaks to the lack of data in this area. Considering that strength gains and hypertrophy tend to slow down or even plateau following long‐term resistance training, training to failure could be an effective means to enhancing strength and muscle mass in trained individuals (Zatsiorsky & Kraemer, 2006). There is counterevidence to this notion, however, since available evidence from recent meta‐analyses suggests that strength and hypertrophy dose–response relationships exist between the number of weekly sets completed (Baz‐Valle et al., 2021; Ralston et al., 2017; Schoenfeld et al., 2017), with trivial differences existing when comparing failure versus non‐failure training (Refalo et al., 2022). Thus, it remains unclear as to whether resistance training to failure optimizes training adaptations in previously trained individuals.

While training at or near failure may negatively affects motor unit characteristics and lead to suboptimal strength outcomes (Carroll et al., 2018; Haun et al., 2017; Nobrega & Libardi, 2016), there are limited data in this area as well. Nobrega et al. (2018) reported that higher‐intensity failure versus non‐failure unilateral leg extensor resistance training over a 12‐week period similarly increased vastus lateralis (VL) electromyography (EMG) amplitudes during maximal isometric knee extensions. A follow‐up study by these researchers similarly indicated that failure versus non‐failure unilateral leg extensor resistance training over a 10‐week period produced similar EMG outcomes (Santanielo et al., 2020). Although these data are insightful, gross EMG assessments do not provide resolution on motor unit characteristics such as individual motor unit recruitment thresholds and/or firing rates.

Given the knowledge gaps discussed above, the purpose of this study was to investigate whether 5 weeks of resistance training near failure (i.e., 0–1 repetitions in reserve per set or “low‐RIR training”) versus ceasing sets 4–6 repetitions short of failure (i.e., “high‐RIR training”) differentially affected strength, hypertrophy, and motor unit characteristics. Herein, resistance‐trained males (*n* = 11) and females (*n* = 8; 24 ± 3 years old, 79.0 ± 22.7 kg, 1.60 ± 0.33 squat: body mass ratio) were randomly assigned to perform 5 weeks (3 days/week) of bench press, back squat, and deadlift resistance training using low‐RIR or high‐RIR guidance per executed set, and both groups performed reduced load training during week 6 (i.e., a deload). Notably, this approach was adopted per the work of Zourdos et al. (2016; Helms et al., 2016). Body composition, proximal/mid/distal VL muscle morphology, strength testing, and VL single motor unit characteristics using surface EMG decomposition were assessed prior to and following the 6‐week program. In accordance with prior literature, we hypothesized that both training paradigms would similarly increase strength and hypertrophy outcomes. Additionally, given the limited EMG data in this area, we assumed single motor unit outcomes would be similar between training groups.

## 2 METHODS

### 2.1 Ethical approval

Experimental procedures were reviewed and approved by the Auburn University Institutional Review Board (IRB approval #: #21‐507 MR 2111) and were conducted according to the standards set by the latest revisions of the Declaration of Helsinki except for the study being registered as a clinical trial.

### 2.2 Physical characteristics

Resistance‐trained college‐aged males (*n* = 11, 24 ± 3 years old, 180.7 ± 6.9 cm, 91.5 ± 22.5 kg; mean ± standard deviation values) and females (*n* = 8, 25 ± 2 years old, 163.4 ± 6.4 cm, 61.9 ± 4.8 kg) consented and completed the study. Participants were screened to ensure they were free of cardio‐metabolic diseases, have not consumed agents that affect hormones within the past 2 months (excluding birth control/oral contraceptives) and disclosed any medications. Participants were considered previously trained if self‐reported training age was greater than or equal to 1 year of resistance training twice weekly. Participants were asked to maintain current nutritional practices and to cease additional training beyond the study.

### 2.3 Experimental protocol

#### 2.3.1 Hydration and body composition assessment

Participants performed a testing battery prior to the start of training (pre), and 48 h following the last resistance training day (post; see Table 1). Notably, these visits were scheduled to be at the same time of day (±1 h). Participants arrived at the laboratory having fasted a minimum of 4 h. Upon arrival, participants submitted a urine sample (~5 mL) for urine specific gravity (USG) assessment. Measurements were performed using a handheld refractometer (ATAGO). USG levels in all participants were ≤1.020, indicating sufficient hydration. Following USG determination, height and weight were attained with a digital column scale (Seca 769), with body mass collected to the nearest 0.1 kg and height to the nearest 0.5 cm.

**TABLE 1:** Training paradigm.

Week 1								
Day 1			Day 2			Day 3		
Exercise	Weight	S × R	Exercise	Weight	S × R	Exercise	Weight	S × R
Squat	70%	3×^a^	Bench	70%	3×^a^	Deadlift	70%	3×^a^
Deadlift	65%	3×^a^	Squat	65%	3×^a^	Bench	65%	3×^a^
RFESS	60%	3 × 15	Low‐incline bench	60%	3 × 15	OHP	60%	3 × 15
RDL	60%	3 × 15	Lat pulldown	60%	3 × 15	BB row	60%	3 × 15
Face‐pull	60%	3 × 15	Goblet squat	60%	3 × 15	BB curl	60%	3 × 15
Skull crushers	60%	3 × 15				Lat raises	60%	3 × 15

Week 2								
Day 4			Day 5			Day 6		
Exercise	Weight	S × R	Exercise	Weight	S × R	Exercise	Weight	S × R
Squat	77.5%	5×^a^	Bench	77.5%	5×^a^	Deadlift	77.5%	5×^a^
Deadlift	70%	3×^a^	Squat	70%	3×^a^	Bench	70%	3×^a^
RFESS	65%	3 × 12	Low‐incline bench	65%	3 × 12	OHP	65%	3 × 12
RDL	65%	3 × 12	Lat pulldown	65%	3 × 12	BB row	65%	3 × 12
Face‐pull	65%	3 × 12	goblet squat	65%	3 × 12	BB curl	65%	3 × 12
Skull crushers	65%	3 × 12				Lat raises	65%	3 × 12

Week 3								
Day 7			Day 8			Day 9		
Exercise	Weight	S × R	Exercise	Weight	S × R	Exercise	Weight	S × R
Squat	85%	4×^a^	Bench	85%	4×^a^	Deadlift	85%	4×^a^
Deadlift	75%	3×^a^	Squat	75%	3×^a^	Bench	75%	3×^a^
RFESS	70%	3 × 10	Low‐incline bench	70%	3 × 12	OHP	70%	3 × 12
RDL	70%	3 × 12	Lat pulldown	70%	3 × 12	BB row	70%	3 × 12
Face‐pull	70%	3 × 12	Goblet squat	70%	3 × 12	BB curl	70%	3 × 12
Skull crushers	70%	3 × 12				Lat raises	70%	3 × 12

Week 4								
Day 10			Day 11			Day 12		
Exercise	Weight	S × R	Exercise	Weight	S × R	Exercise	Weight	S × R
Squat	90%	5×^a^	Bench	90%	5×^a^	Deadlift	90%	5×^a^
Deadlift	80%	3×^a^	Squat	80%	3×^a^	Bench	80%	3×^a^
RFESS	75%	3 × 8	Low‐incline bench	75%	3 × 8	OHP	75%	3 × 8
RDL	75%	3 × 8	Lat pulldown	75%	3 × 8	BB row	75%	3 × 8
Face‐pull	75%	3 × 8	Goblet squat	75%	3 × 8	BB curl	75%	3 × 8
Skull crushers	75%	3 × 8				Lat raises	75%	3 × 8

Week 5								
Day 13			Day 14			Day 15		
Exercise	Weight	S × R	Exercise	Weight	S × R	Exercise	Weight	S × R
Squat	95%	6×^a^	Bench	95%	6×^a^	Deadlift	95%	6×^a^
Deadlift	85%	3×^a^	Squat	85%	3×^a^	Bench	85%	3×^a^
RFESS	80%	3 × 6	Low‐incline bench	80%	3 × 6	OHP	80%	3 × 6
RDL	80%	3 × 6	Lat pulldown	80%	3 × 6	BB row	80%	3 × 6
Face‐pull	80%	3 × 6	Goblet squat	80%	3 × 6	BB curl	80%	3 × 6
Skull crushers	80%	3 × 6				Lat raises	80%	3 × 6

Week 6							
Day 16			Day 17			Day 18	
Exercise	Weight	S × R	Exercise	Weight	S × R	Exercise	Note
Squat	65%	3 × 3	Squat	65%	3 × 3	Squat	Strength testing for these variables
Deadlift	65%	3 × 3	Deadlift	65%	3 × 3	Deadlift	
Bench	65%	3 × 3	Bench	65%	3 × 3	Bench	

Abbreviations: BB, barbell; OHP, overhead press; RDL, Romanian deadlifts; RFESS, rear foot elevated split squats; RIR, repetitions in reserve; S × R: number of sets and repetitions.

^a^ Exercises low‐RIR group took each set to perceived fatigue (i.e., RIR of 0–1), and high‐RIR group took each set to 4–6 RIR (i.e., 4–6 RIR). All other exercises were performed as programed between the low‐RIR and high‐RIR groups, and Week 6 exercises were performed as programmed in both groups to serve as a deload week.

#### 2.3.2 Ultrasonography for VL characteristics

Ultrasound derived VL muscle cross‐sectional area (mCSA) was obtained as previously described by our laboratory (Ruple et al., 2022). Briefly, real‐time B‐mode ultrasonography (NextGen LOGIQe R8; GE Healthcare) utilizing a multi‐frequency linear‐array transducer (L4‐12T, 4–12 MHz; GE Healthcare) was used to capture VL mCSA at the proximal, mid, and distal portion of the right leg. Locations were determined by measuring the total distance from the mid‐inguinal crease in a straight line to the proximal patella, with the hip and knee flexed at 90°, then making a mark at the location corresponding to 33%, 50%, and 67% of the total length. From that location, a permanent marker was used transversely to mark the VL at each region. After the participant waited a minimum of 5 min of supine rest, a flexible, semi‐rigid pad was placed around the thigh and secured with an adjustable strap. The pad was used solely as a guide to ensure probe movement in the transverse plane starting at the lateral aspect of the VL moving medially until the rectus femoris was visualized. VL mCSA images were captured using the panoramic function of the device (LogicView; GE Healthcare). All ultrasound settings were held constant across participants and laboratory visits (frequency: 10 MHz, gain: 50 dB, dynamic range: 75). Images were downloaded and analyzed off‐line using the freely available ImageJ software (National Institutes of Health). VL mCSA was calculated by manually tracing the border of the VL using the polygon function, with care taken to exclude any connective tissue within the region of interest. All ultrasound images were captured and analyzed by the same investigators at each timepoint (KCY and JSG, respectively). Previously determined test–retest reliability for VL mCSA yielded an intra‐class correlation coefficient (ICC_3,1_) of 0.99, standard error of the measurement of 1.57 cm^2^, and minimal difference of 3.08 cm^2^.

#### 2.3.3 Isometric knee extensor testing

Participants were seated and restrained on a dynamometer chair (System 4 Pro; BioDex Medical Systems) for isometric assessment of the isolated knee joint. The seat was adjusted so that the right knee aligned with the axis of the dynamometer's lever arm, and the hip was positioned at 90°. Straps were placed and secured across both shoulders, hips, and contralateral leg. Another strap was tightly fastened around the right leg, ~2–3 cm above the ankle, which was attached to the dynamometer for measurement of isometric force. Prior to testing, participants performed a warm‐up set that consisted of three, 10‐s, isometric contractions corresponding to 50%, 70%, and 90% of perceived max. Following the warm‐up, participants performed three, 3‐s maximum voluntary contractions (MVCs), separated by 3 min of rest. The highest MVC recorded was noted and used for subsequent tests. Following determination of the MVC, participants performed two trapezoidal isometric contractions at 80% of the MVC in accordance with a visual template. Participants increased isometric force from 0% to 80% MVC in 8 s, held 80% MVC constant for 10‐s, and decreased force from 80% to 0% MVC in 8 s. Participants were allotted 3 min of rest between tests.

#### 2.3.4 EMG signal recording

Surface EMG signals were recorded from the VL with a Bagnoli 16‐channel desktop system (Delsys, Inc.). The signals were detected with a 5 × 5 mm, surface array EMG sensor (Delsys, Inc.) that consists of five pin electrodes (Nawab et al., 2010). Four of the five electrodes are oriented in a square, with the fifth electrode located in the center of the four‐pin square and at a fixed distance of 3.6 mm from the other electrodes. Prior to detecting EMG signals, the skin over the VL and patella was shaved and cleansed with rubbing alcohol. Oil, debris, and dead skin cells were removed with hypo‐allergenic tape, and a reference electrode was placed over the patella. The sensor was secured to the VL muscle with adhesive tape and was in accordance with the procedure outlined by Zaheer et al. (2012). Pairwise subtraction of the five electrodes was used to derive four single differential EMG channels. The EMG signals acquired were differentially amplified and filtered with a bandwidth of 20 to 450 Hz. EMG signals obtained from the participants were sampled at 20 kHz. Prior to data collection, a signal‐to‐noise ratio >3.0 and line interference <1.0 were ensured during a 20% MVC force surface EMG signal quality check.

#### 2.3.5 EMG decomposition and spike trigger averaging

Following data acquisition, the four separate filtered surface EMG signals from the VL were decomposed into their constituent motor unit action potential trains using the Precision Decomposition III (PD III) algorithm described by De Luca et al. (2006) and further expanded upon by Nawab et al. (2010). Once the signals were successfully decomposed, the reconstruct‐and‐test procedure was used to determine the accuracy of each motor unit (De Luca & Contessa, 2012). Motor units identified at the >90% accuracy threshold level were included for further analysis.

The output from the PD III algorithm consisted of firing times and four unique waveforms corresponding to four bipolar channels from the four electrode pairings for each motor unit. Using the PD III derived firing times of the identified motor units at the >90% accuracy threshold, the spike trigger averaging (STA) procedure was performed on each surface EMG signal, resulting in four representative STA motor unit action potential waveforms for each motor unit. The coefficient of variations of the peak‐to‐peak amplitudes from the STA motor unit action potential waveforms were calculated over time. The window length was 4 s and shifted over the surface of EMG signal using a step size of 1 s. The maximum correlation coefficients between STA and PD III derived waveforms were also calculated. For the STA motor unit action potential waveforms, the composite waveform created from the firing events was compared to the waveforms generated by the PD III algorithm (Hu et al., 2013a, 2013b; McManus et al., 2015). Motor units selected for the reliability and agreement statistics when using STA must have had a correlation coefficient >0.7 and coefficient of variation <0.3 across all four channels (Hu et al., 2013a; McManus et al., 2016). Secondary analyses included the removal of motor units that did not meet these criteria based on the STA procedure. For additional information on this verification procedure, the reader is directed to the work of Herda et al. (2020).

For each motor unit, two parameters (recruitment threshold [%MVC] and mean firing rate [pps]) were extracted at the targeted contraction level. Each motor unit's recruitment threshold was quantified as the percentage of the MVC corresponding to its first firing during the ascending portion of the trapezoidal contraction. Each motor unit's time‐varying mean firing rate was computed by passing each train of firings though a 1 s Hanning window. Linear regressions were applied on a participant‐by‐participant basis to calculate the slopes (pps/%MVC) and *y*‐intercepts (pps) for the PD III derived relationships. Linear regressions were only applied to included motor units that could be accurately identified by both PDIII and STA procedure at the >90% accuracy threshold level. Our previous work has highlighted the linearity and appropriateness of using these relationships to investigate individual motor unit data (Harmon et al., 2019).

#### 2.3.6 Maximal strength testing

Following isometric testing, participants were escorted to the testing facility, where they started strength testing for barbell back squat, barbell bench press, and barbell deadlift. Each exercise began with warm‐up sets starting with 50% one‐repetition maximum (1RM) for 10 repetitions, followed by 75% 1RM for five repetitions, and finally ending the warm‐up with 90% 1RM for three repetitions. All calculations were based on 1RM projections that were self‐selected by the participants. Following the warm‐ups, participants performed their self‐selected 1RM. Weight was increased by 5%–10% until 1RM was achieved. Strength testing was supervised by investigators with National Strength and Conditioning Association (NSCA) Certified Strength and Conditioning Specialist credential (C.L.S., M.A.S, and M.C.M.).

### 2.4 Resistance training program

Participants were randomly assigned (based on Wilks score) to one of two groups. A Wilks score normalizes strength across different bodyweights and sex. The low‐RIR group was instructed to perform barbell back squat, barbell bench press, and barbell deadlift as close to volitional failure as possible (i.e., RIR of 0–1). The high RIR group was instructed to perform squat, bench press, and deadlift as prescribed on the training sheet, and adjusting the load appropriately to not reach volitional fatigue throughout the study (i.e., RIR 4–6; see Table 1). As stated in the Introduction, the RIR approach mimicked that described by Zourdos et al. (2016; Helms et al., 2016). Participants completed a full‐body training, three times per week, for 5 weeks, and a 1‐week deload that consisted of squat, bench, and deadlift at a reduced training volume. Squat, bench, and deadlift exercises were performed twice a week each and were the only low‐RIR exercises performed near failure. All other accessory lifts were performed once a week with equalized higher RIR values between groups, participants were given recommended loads based on %1RM predictions but ultimately chose loads appropriate to perform the repetitions at prescribed intensities.

There was no direct supervision of sessions. Participants performed training in local gymnasiums and were required to log training on a specific Google Sheets document that was only accessible by the participant and study coordinator. Notably, these sheets were inspected daily by the study coordinators (B.A.R. and M.A.S.), and participants that lagged in filling sheets out were contacted via phone or email to remind them to update their online training log. Participants were instructed to record RIR after each exercise. Additionally, the study coordinator communicated with participants multiple times on a weekly basis to ensure that training was proceeding according to prescribed loads.

### 2.5 Statistical analyses

All statistical analyses were performed using SPSS Version 25 (IBM SPSS Statistics Software). Prior to analysis, assumptions testing for normality were performed using Shapiro–Wilk's test for all baseline dependent variables at pre and all variables were normally distributed, except for mid mCSA. Given that the majority of data were normally distributed, all data were analyzed using parametric statistical tests. Two‐way, condition × time (C × T), repeated measure ANOVAs were used to determine changes in dependent variables over time. If a significant C × T interaction was observed, then within‐group dependent samples *t*‐tests were performed to examine pre versus post for each group, and an independent samples *t*‐test was used for comparison between groups at pre and post. Other dependent variables were analyzed using dependent samples *t*‐tests or analyzed as described in prior sections (i.e., linear regression for motor unit characteristics). Eta square (*η* ^2^) effect sizes are also provided for certain between‐group comparisons and interactions, and effect size ranges were classified as follows: <0.06 = small effect, 0.06–0.14 moderate effect, and >0.14 large effect. Finally, Pearson correlations were performed on select variables. All data in tables and figures are presented as the mean ± standard deviation (SD) values, and statistical significance was set at *p* < 0.05.

## 3 RESULTS

### 3.1 Baseline characteristics

There were no baseline differences between training groups for select dependent variables, which are displayed in Table 2. Self‐reported adherence over the 6‐week training period was 100% for all participants as assessed through training logs and persistent communication throughout the study.

**TABLE 2:** Baseline characteristics between groups.

	low‐RIR	high‐RIR	*p*‐Value
Participants	10	9	—
Sex (M/F)	6/4	5/4	—
Age (years)	25 ± 3	24 ± 2	0.77
Height (cm)	173 ± 13	174 ± 8	0.88
Body mass (kg)	78.7 ± 26.7	79.4 ± 18.9	0.94
Body fat (%)	17.1 ± 9.2	15.9 ± 9.9	0.78
Squat 1RM (kg)	127 ± 45	125 ± 41	0.92
Bench 1RM (kg)	84 ± 43	80 ± 44	0.85
Deadlift (kg)	137 ± 48	143 ± 42	0.76
Isometric MVC (N·m)	285 ± 83	277 ± 91	0.84

*Note*: Values are presented as mean ± SD. All values are from pre measurements.

Abbreviations: 1‐RM, one‐repetition maximum; cm, centimeters; F, females; high‐RIR, group training further from failure per set for exercises indicated in Table 1 (4–6 RIR); kg, kilograms; low‐RIR, group training closer to failure per set for exercises indicated in Table 1 (0–1 RIR); M, males; MVC, maximum voluntary contraction; N·m, Newton‐meters; RIR, repetitions in reserve.

Notably, all 19 participants self‐reported resistance training for 1 year or greater (6 ± 4 years, range = 1–14 years). The men in the study possessed body mass‐normalized bench press and back squat strength values of 1.27 ± 0.23 and 1.74 ± 0.27, respectively, which (relative to prior literature) is in line with men who have prior resistance training experience (Hoeger et al., 1990; Shimano et al., 2006). The women in the study possessed body mass‐normalized bench press and back squat strength values of 0.64 ± 0.16 and 1.40 ± 0.32, respectively, which again aligns with women possessing prior resistance training experience (Hoeger et al., 1990; Norum et al., 2020).

### 3.2 Training volume and RIR

**FIGURE 1:**
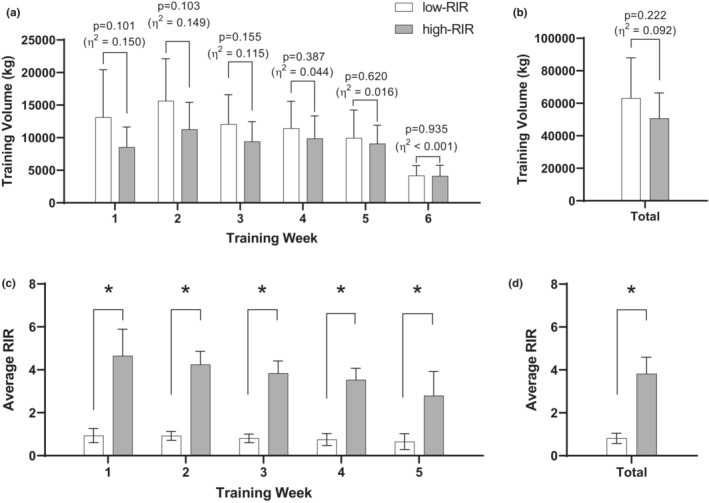
Training volume and RIR. Data in panel (a) illustrate week‐by‐week training volume (for squat, bench press, and deadlift only) between training conditions. Data in panel (b) illustrate total training volume over the 6‐week period (for squat, bench press, and deadlift only) between training conditions. Data in panel (c) illustrate week‐by‐week RIR reported by participants (for squat, bench press, and deadlift only) between training conditions *p* < 0.001). Data in panel (d) illustrate total averaged RIR over the 6‐week period (for squat, bench press, and deadlift only) between training conditions. *n* = 10 low‐RIR and *n* = 9 high‐RIR participants for panels (a, b), and *n* = 10 low‐RIR and *n* = 8 high‐RIR participants for (c) and (d). high‐RIR, group training further from failure per set for exercises indicated in Table 1 (4–6 RIR); low‐RIR, group training closer to failure per set for exercises indicated in Table 1 (0–1 RIR); RIR, repetitions in reserve.

Training volume during the intervention did not differ between groups at any week (*p* > 0.05, Figure 1a), or overall (low‐RIR: 63,148 ± 24,827 kg, high‐RIR: 50,707 ± 15,708 kg, *p* = 0.222, Figure 1b). However, large effects were during Weeks 1 and 2 observed whereby low‐RIR lifted more weight than high‐RIR, and a moderate effect was evident for total training volume. The average RIR for the low‐RIR was significantly lower than that for high‐RIR group each week (*p* < 0.001, Figure 1c), as well as overall average (low‐RIR: 0.81 ± 0.24, high‐RIR: 3.82 ± 0.77, *p* < 0.001, Figure 1d).

### 3.3 Muscle strength adaptations

However, there were no C × T interactions for squat 1RM (low‐RIR: 127 ± 45 kg to 138 ± 46 kg, high‐RIR: 125 ± 41 kg to 134 ± 42 kg, *p* = 0.159, *η* ^2^ = 0.113), bench press 1RM (low‐RIR: 84 ± 43 kg to 88 ± 43 kg, high‐RIR: 80 ± 44 kg to 84 ± 41 kg, *p* = 0.818, *η* ^2^ = 0.03), and deadlift 1RM (low‐RIR: 137 ± 48 kg to 144 ± 47 kg, high‐RIR: 144 ± 42 kg to 151 ± 46 kg, *p* = 0.756, *η* ^2^ = 0.006). However, there were significant main effects of time for squat 1RM (126 ± 42 kg to 136 ± 43 kg, *p* < 0.001), bench press 1RM (82 ± 42 kg to 86 ± 41 kg, *p* < 0.001), and deadlift 1RM (141 ± 45 kg to 147 ± 45 kg, *p* = 0.020).

**FIGURE 2:**
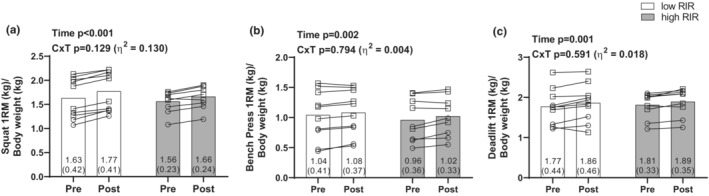
Muscle strength adaptations. All figures show pre and post values for respective lifts, divided by the individuals body mass (kg). (a) Back squat, (b) bench press, and (c) deadlift. Values are presented as mean ± SD. Individual responses also illustrated, with open circles indicating females, and open squares indicating males. Pre, 7 days before the 6‐week training intervention. Post, 48 h following the last training bout. *n* = 10 low‐RIR and *n* = 9 high‐RIR participants in all panels. 1RM, one‐repetition maximum; C × T, condition by time interaction; high‐RIR, group training further from failure per set for exercises indicated in Table 1 (4–6 RIR); low‐RIR, group training closer to failure per set for exercises indicated in Table 1 (0–1 RIR); RIR, repetitions in reserve.

To account for potential sex differences, strength metrics were also normalized by body mass. Again, no C × T interactions existed for relative squat strength (low‐RIR: 1.63 ± 0.42 to 1.77 ± 0.41, high‐RIR: 1.56 ± 0.23 to 1.66 ± 0.24, *p* = 0.129, *η* ^2^ = 0.130), relative bench press strength (low‐RIR: 1.04 ± 0.41 to 1.08 ± 0.37, high‐RIR: 0.96 ± 0.36 to 1.02 ± 0.33, *p* = 0.794, *η* ^2^ = 0.004), or relative deadlift strength (low‐RIR: 1.77 ± 0.44 to 1.86 ± 0.46, high‐RIR: 1.81 ± 0.33 to 1.89 ± 0.35, *p* = 0.591, *η* ^2^ = 0.018). However, again, there were significant main effects of time for relative squat strength (1.60 ± 0.33 to 1.72 ± 0.33, *p* < 0.001, Figure 2a), relative bench press strength (1.00 ± 0.38 to 1.05 ± 0.35, *p* = 0.002, Figure 2b), and relative deadlift strength (1.79 ± 0.38 to 1.87 ± 0.40, *p* = 0.001, Figure 2c).

### 3.4 Ultrasound‐derived mCSA

**FIGURE 3:**
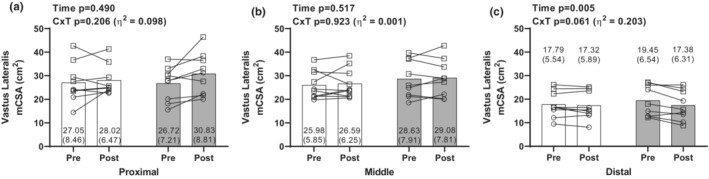
Changes in VL mCSA. All figures show pre and post values for mCSA values of the VL from the (a) proximal (33%), (b) middle (50%), and (c) distal (67%) portion of the femur. As indicated in the Figure 2 legend, white bars are the low‐RIR group and gray bars are the high‐RIR group. Values are presented as mean ± SD. Individual responses also illustrated, with open circles indicating females, and open squares indicating males. Pre, 7 days before the 6‐week training intervention. Post, 48 h following the last training bout. *n* = 9 low‐RIR and *n* = 9 high‐RIR participants in panels (a, c), and *n* = 10 low‐RIR and *n* = 9 high‐RIR participants in panel (b). C × T, condition by time interaction; high‐RIR, group training further from failure per set for exercises indicated in Table 1 (4–6 RIR); low‐RIR, group training closer to failure per set for exercises indicated in Table 1 (0–1 RIR); mCSA, muscle cross‐sectional area; RIR, repetitions in reserve; VL, vastus lateralis.

Pre and post intervention mCSA changes were measured at the three locations along the VL. No significant main effects of condition, time, or C × T interactions existed for proximal VL mCSA (Figure 3a) or mid‐thigh VL mCSA changes (Figure 3b). There was a time effect for distal VL mCSA (*p* = 0.005), and while there was not a significant C × T interaction (*p* = 0.061, *η* ^2^ = 0.203, Figure 3c), the interaction effect size was large.

In an attempt to determine why a high degree of individual response heterogeneity existed for the site‐specific VL mCSA values, we performed various correlations. Non‐significant, negative associations existed between training age and the percent changes in proximal VL mCSA (*r* = −0.292, *p* = 0.239), mid‐thigh VL mCSA (*r* = −0.266, *p* = 0.287), and distal VL mCSA (*r* = −0.392, *p* = 0.108). Hence, training age showed poor associations with these muscle morphology changes. Interestingly, a significant, positive correlation existed between the percent changes in proximal and mid‐thigh VL mCSA (*r* = 0.605, *p* = 0.008). However, the percent change in distal VL mCSA did not significantly correlate with the percent changes in the proximal (*r* = 0.419, *p* = 0.083) or mid‐thigh sites (*r* = 0.124, *p* = 0.625).

### 3.5 Isometric peak torque and VL motor unit adaptations

**FIGURE 4:**
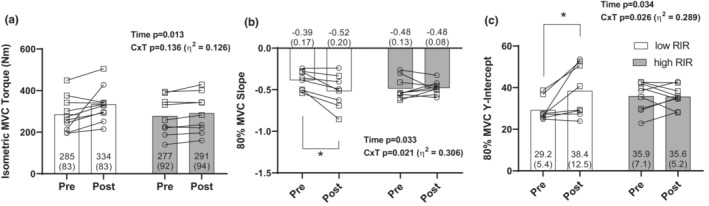
Motor unit data. All figures show pre and post values. Data in panel (a) illustrate isometric MVC torque. Data in panel (b) illustrate the 80% MVC slope. Data in panel (c) illustrate 80% MVC *y*‐intercept. Values are presented as mean ± SD. (*p < 0.05). As indicated in the Figure 2 legend, white bars are the low‐RIR group and gray bars are the high‐RIR group. Individual responses also illustrated, with open circles indicating females, and open squares indicating males. Pre, 7 days before the 6‐week training intervention. Post, 48 h following the last training bout. Data are for *n* = 10 low‐RIR and *n* = 9 high‐RIR participants for panel (a), and *n* = 8 low‐RIR and *n* = 9 high‐RIR participants for all other panels. C × T, condition by time interaction; high‐RIR, group training further from failure per set for exercises indicated in Table 1 (4–6 RIR); low‐RIR, group training closer to failure per set for exercises indicated in Table 1 (0–1 RIR); MVC, maximal voluntary contraction of the knee extensors; RIR, repetitions in reserve.

Although there was not a significant C × T interaction for isometric MVC torque, there was a medium‐to‐large effect size for this variable (low‐RIR: 285 ± 83 N·m to 334 ± 83 N·m, high‐RIR: 277 ± 92 N·m to 291 ± 94 N·m, *p* = 0.136, *η* ^2^ = 0.126, Figure 4a), and a significant main effect of time was also observed (282 ± 85 N·m to 313 ± 89 N·m, *p* = 0.013).

**FIGURE 5:**
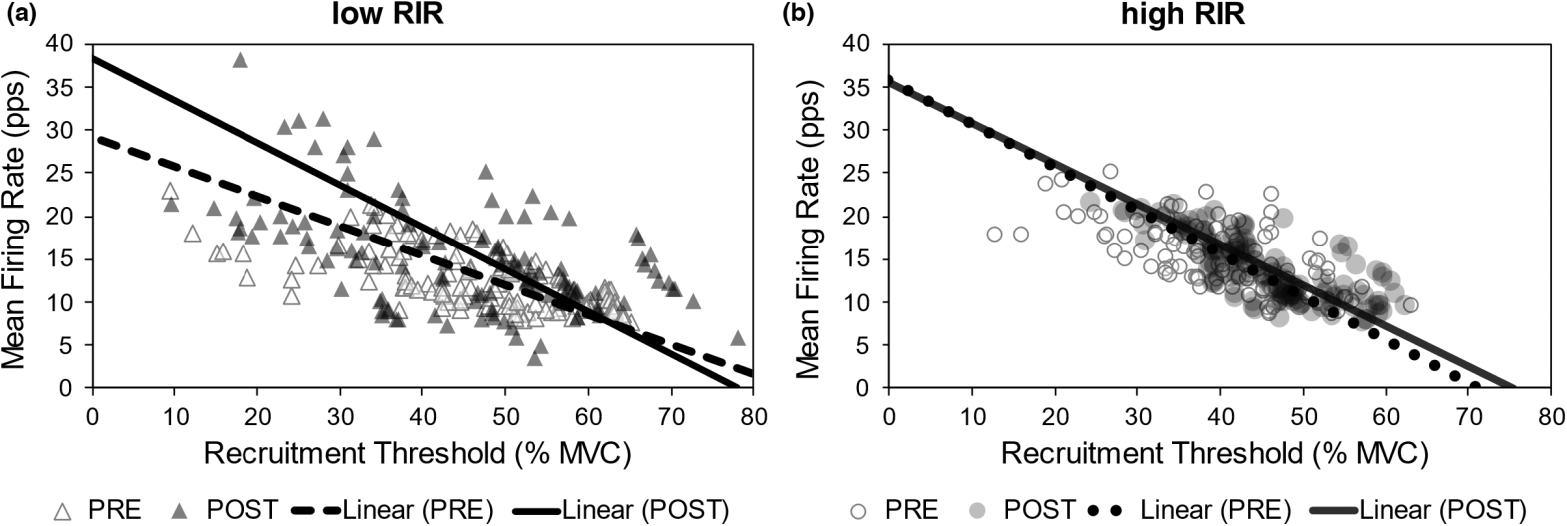
VL motor unit mean firing rate versus recruitment threshold relationships. Both figures show pre and post VL motor unit data, displaying the linear relationships between mean firing rates (*y*‐axes) and recruitment thresholds (*x*‐axes). The linear regression lines represent the mean slopes and *y*‐intercepts derived from each time point. Whereas the data appears stable for high‐RIR, note the upward shift of the linear regression line for low‐RIR. high‐RIR, group training further from failure per set for exercises indicated in Table 1 (4–6 RIR); low‐RIR, group training closer to failure per set for exercises indicated in Table 1 (0–1 RIR); MVC, maximal voluntary contraction of the knee extensors; RIR, repetitions in reserve; VL, vastus lateralis.

Significant C × T interactions were present for the slope and *y*‐intercept for the mean firing rate versus recruitment threshold relationship at the 80% MVC torque level (slope: *p* = 0.021, *η* ^2^ = 0.306; *y*‐intercept: *p* = 0.026, *η* ^2^ = 0.289, Figure 4b,c). In the low‐RIR group, the post slope values were significantly less (i.e., more negative) than the pre slope values, and the post *y*‐intercept values were significantly higher than *y*‐intercept values acquired at pre. Pre and post slope and *y*‐intercept values were consistent in the high‐RIR group. Regressed motor unit recruitment threshold and firing rate relationships for each condition are illustrated in Figure 5a,b. Notably 17 ± 9 and 15 ± 2 motor units were quantified for analysis in the low‐RIR group prior to and following the intervention, and 14 ± 5 and 16 ± 6 motor units were quantified for analysis in the high‐RIR group at these time points.

## 4 DISCUSSION

This study investigated the effects of low‐RIR versus high‐RIR training on strength, hypertrophy, and motor unit adaptations in previously trained males and females. Strength outcomes and changes in proximal and mid VL mCSA occurred between groups. Surprisingly, a decrease in distal VL mCSA was observed (i.e., a significant main effect of time), but no significant interaction was observed between training groups. Regarding neural adaptations, differences between conditions were observed for slope and *y*‐intercept, with the low‐RIR group significantly decreasing slope values while also increasing *y*‐intercept values after the training intervention. These latter data indicate an increase in mean firing rates for the low‐threshold motor units in the low‐RIR group. Details of each outcome are discussed sequentially herein.

Given that the high‐RIR group performed training further from failure, volume‐load (i.e., sets × repetitions × load per repetition; Figure 1) would be expected to favor the low‐RIR group. However, while there were weekly differences in volume‐load that favored low‐RIR as seen in Figure 1, especially in weeks 1 and 2 where the effect sizes between groups were large, these differences did not reach statistical significance. Although this finding was unexpected, there are reasonable explanations. First, assigned lifting loads for the bench, barbell squat and deadlift exercises were relatively lower in the first 2 weeks of training (i.e., 65%–77.5% initial 1RM), whereas loads became greater during the next 3 weeks of training (i.e., 75%–95% initial 1RM). Hence, this lead to enough of a between‐group divergence in volume‐load to yield large effect sizes in weeks 1–2, whereas the effects size small‐to‐moderate during weeks 3–5 and when analyzing the total combined volumes between groups. As can be seen in Figure 1, this led to a reduction in volume‐load in general. Interestingly, Carroll et al. (2018) similarly reported that resistance training to failure over a 10‐week period (i.e., “RM” training) yielded a similar total volume‐load relative to a group of participants who did not train to failure (i.e., relative intensity training, or “RI” training). Outcomes from our study and the aforementioned investigation may be due to sets taken close to failure in the low‐RIR group inducing more fatigue and necessitating a decrease in repetitions to maintain load. Alternatively stated, it is likely that the progression from moderate to higher loads reduced the ability to maintain volume‐load differences between groups.

Back squat, deadlift, and bench press strength metrics similarly increased in both groups, and these results are in line with a meta‐analysis by Grgic and colleagues suggesting that training to failure in previously trained individuals (shown in their subgroup analysis) is not necessary to maximize strength gains (Grgic et al., 2022). While isometric MVC torque increased in both groups as evidenced by a significant main effect of time, the moderate‐to‐large effect size observed in the interaction points to a greater within‐group increase in the low‐RIR group (17.2%) than in the high‐RIR group (5.1%). These collective data largely contrast Carroll et al. (2018) who reported that 10 weeks of RM training led to suboptimal isometric mid‐thigh pull strength increases relative to RI training. Differences between study outcomes could be due to a variety of factors including participant characteristics, the duration of training, and the structure of training. Specifically, Carroll and colleagues examined younger men who presented strength metrics that paralleled collegiate athletes, and while our participants self‐reported resistance training >1 year and presented an average squat: body mass ratio indicative of being well‐trained, it is likely that the participants examined by Carroll et al. were more well‐trained. Carroll and colleagues also utilized a 10‐week supervised training program whereby an advanced periodization approach was adopted, whereas the current low‐ and high‐RIR participants engaged in a 5‐week training block whereby volume‐load (through increases in training weight) was incrementally increased. Hence, it remains possible that we may have observed similar strength outcomes to those reported by Carroll and colleagues had our training design mimicked their paradigm.

There were no significant main effects of time in either group for proximal or middle VL mCSA changes. These data can potentially be explained by the high average training age (6 ± 4 years) for the cohort, as less muscle hypertrophy in well‐trained individuals would be expected given the short duration of the intervention. However, there was a large degree of inter‐individual variability in these outcomes. No significant associations were found to exist between training age and these hypertrophic outcomes as reported in the Results section, and we have published prior data in trained individuals showing that training age does not significantly correlate with hypertrophic outcomes in trained men following 6 weeks of high volume training (Haun et al., 2019). Hence, we are uncertain as to why there were “lower” and “higher” hypertrophic responders, and mechanisms related to this phenomenon (none of which could be determined herein due to lack of muscle biopsies) are still being investigated by various research groups (Roberts et al., 2018). A significant main effect of time for a loss in distal VL mCSA was also observed herein, and while the C × T interaction for this variable was not statistically significant, there was a large effect size observed for the interaction which was driven by a greater decrease in the high‐RIR group. We are uncertain as to how to interpret these data as this may be a spurious finding given that we would not expect a decrease in muscle size at any site from RT. In this regard, Mangine et al. (2018) reported that 8 weeks of high‐load/low volume resistance training in previously trained men increases mCSA values in the same three VL sites that we assessed. However, given that their study implemented high loads and lower volumes throughout, it remains possible that our higher‐volume program led to morphological changes (e.g., pennation angle changes in the middle and more proximal VL sites) that could have driven decreases in distal VL mCSA values. What should also be noted is that the VL site mCSA percentage change associations showed good agreement between the proximal and mid‐thigh sites, whereas the distal site did not significantly correlate with either of the former. Hence, these data support the contention that our distal VL data may have yielded spurious findings. Notwithstanding, these data highlight the need to examine how different resistance training paradigms affect morphology outcomes at multiple VL sites.

Perhaps the most interesting observation herein was the changes in motor unit characteristics in the low‐RIR group. Closer inspection of the regressed recruitment threshold and firing rate relationship in the low‐RIR group after the intervention show a significant decrease in slope values and significant increases in *y*‐intercept values. The trend observed in the low‐RIR group suggests an increase in the firing rates of earlier recruited motor units because of the training intervention. Such findings of non‐uniform changes in firing rates following resistance training have been documented (Watanabe et al., 2018, 2020) and suggest that earlier recruited motor units are more susceptible to increasing their firing rates after resistance training interventions. However, general changes in motor unit firing rates after resistance training remain mixed due to the variability of training interventions and muscle contraction types used to record motor units (Herda, 2022). Given the specific scope of the training intervention, low‐RIR training in the exercises included in this study may influence the firing rates of earlier recruited motor units. The increase in isometric MVC torque in the low‐RIR group (as determined by the moderate‐to‐high effect size of the interaction reported in Figure 4a) may also be explained, in part, by this non‐uniform increase in motor unit firing rates. As the present study was the first of its kind, additional research is needed to confirm the extent to which low‐ and high‐RIR training may influence the voluntary control of motor units.

There are limitations to consider with the current study. Our study had a total of 19 participants, which limited our statistical power. Another significant limitation is that our study was unsupervised. While we did employ a training log and weekly check‐ins, it is within the realm of possibility that participants were not truthful when logging their training sessions. Furthermore, there may have been unintended effects due to changes in nutrition between groups, as nutrition was not tracked or directly prescribed beyond encouraging participants to continue to practice normal dietary habits. However, there were very few changes in weight (or body composition) suggesting that there were not large differences between caloric consumption above or below maintenance between groups. Our study was also only 6 weeks in duration (5 weeks of progressive overload and a 1‐week deload), thus perhaps not allowing enough time for hypertrophy adaptations to occur in a well‐trained population. We also decided a priori that implementing a deload week for both groups prior to post‐intervention testing was practical given that advanced resistance training programs implement a deload week between training blocks (Ogasawara et al., 2011). However, we did miss the early adaptations to each style of training by not performing post‐intervention testing after the 5‐week time point, and this too is a limitation. As mentioned previously, the motor unit adaptations observed in this study are limited to the type of exercise intervention administered, the contraction type used to record motor units, and the type of muscle analyzed. Another limitation herein is that the females were enrolled without considering menstrual cycle phase given that this was not a primary interest in the current study. However, the reader should be aware that several studies have how menstrual cycle phase‐based training affects resistance training outcomes (Reis et al., 1995; Sakamaki et al., 2012; Sakamaki‐Sunaga et al., 2016; Sung et al., 2014; Thompson et al., 2021), and a recent perspective suggests follicular phase‐based resistance training (vs. luteal phase‐based training) may be optimal in this regard (Kissow et al., 2022). Finally, while it is possible that RIR estimates were inaccurate, there is evidence suggesting experienced individuals are accurate in RIR predictions, particularly at moderate repetition ranges and closer to failure (Zourdos et al., 2021).

## 5 CONCLUSIONS

These data are informative for recreationally trained individuals given that performing moderate to higher resistance loads (i.e., 65%–95% 1RM) using 0–1 versus 4–6 repetitions in reserve following each set promotes similar increases in strength. A recent report by Refalo et al. indicates that performing an acute bout of resistance exercise at or close to failure (0–1 RIR) leads to poorer post‐exercise recovery and worsened muscle soreness and general feelings of well‐being compared to higher RIR training (3 RIR; Refalo et al., 2023). Hence, high‐RIR training is seemingly an effective method for increasing strength in recreationally trained individuals during a 5‐ to 6‐week training block while not overtaxing the trainee. However, low‐RIR training seemingly increases the firing rates of earlier recruited motor units and further research is needed to determine whether this training adaptation has practical relevance.

## AUTHOR CONTRIBUTIONS

Bradley A. Ruple conceived study, consent, recruitment, contact with participants throughout, data collection, drafted methods of manuscript, drafted figures, critically edited draft, approved final draft. Daniel L. Plotkin primarily drafted manuscript, assisted with figures, approved final draft. Morgan A. Smith involved in recruitment, contact with participants throughout, data collection, critically edited draft, approved final draft. Joshua S. Godwin, Mason C. McIntosh, Nicholas J. Kontos, Cleiton A. Libardi, Kaelin C. Young, and Casey L. Sexton involved in data collection, critically edited draft, approved final draft. Jonathan P. Beausejour, Juan P. Rodriguez, and Jason I. Pagan involved in EMG data collection, critically edited draft, approved final draft. Daniel Sheldon and Kevan S. Knowles involved in EMG data analysis, critically edited draft, approved final draft. Matt S. Stock conceived EMG aspect of study, EMG data collection, EMG data analysis, drafted methods of manuscript, critically edited draft, approved final draft. Michael D. Roberts conceived the study, established collaborations between multiple research laboratories for this project, assisted with data collection, critically edited draft, approved final draft.

## FUNDING INFORMATION

No funding was procured for this study. M.C. McIntosh was fully supported through a T32 NIH grant (T32GM141739). C. A. Libardi was supported by the São Paulo Research Foundation (n° 2020/13613‐4) and Technological Development (n° 302801/2018‐9).

## CONFLICT OF INTEREST STATEMENT

In relation to the current data, the authors declare that no conflicts of interest exist.
